# A bibliometric analysis of traditional Chinese non-pharmacological therapies in the treatment of knee osteoarthritis from 2012 to 2022

**DOI:** 10.3389/fnins.2023.1097130

**Published:** 2023-03-01

**Authors:** Shouyao Zhang, Yuanwang Wang, Meng Zhou, Shan Jia, Ye Liu, Xinghe Zhang, Xiantao Tai

**Affiliations:** School of Second Clinical Medicine, Yunnan University of Chinese Medicine, Kunming, China

**Keywords:** non-pharmacological therapy, traditional Chinese medicine, bibliometric, knee osteoarthritis, knowledge map

## Abstract

**Objective:**

The benefits of traditional Chinese non-pharmacological therapies in the treatment of Knee osteoarthritis (KOA) are receiving increasing attention. Therefore, this study aims to systematically analyze the global research on the treatment of KOA by Chinese traditional non-pharmacological therapies using bibliometric analysis and present the results with a knowledge map form.

**Methods:**

Literature related to traditional Chinese non-pharmacological therapies used in the treatment of KOA from 2012 to 2022 was searched from the Web of Science core database and PubMed database. CiteSpace, SCImago Graphica and VOSviewer were used to extract nations, institutions, journals, authors, references, keywords, as well as the most widely used acupoints, therapies and evaluation indexes.

**Results:**

A total of 375 literature have been included. 32 countries around the world have participated in the research. China, the United States, and Europe were at the center of the global cooperation network. The most prolific institutions and authors were from China represented by Cun-zhi Liu and Jian-feng Tu of Beijing University of Chinese Medicine, the institution with the highest cited frequency was University of York, and “Osteoarthritis Cartilage” was the most frequently cited journal. The most frequently cited literature was “OARSI guidelines for the non-surgical management of knee, hip, and poly articular osteoarthritis.” 22 kinds of Chinese non-pharmacological therapies were used to treat KOA, among which acupuncture was the most commonly used one, and ST36 (Zusanli) and WOMAC were the most commonly selected acupoint and evaluation index.

**Conclusion:**

In the past decade, the value of Chinese non-pharmacological therapies in the treatment of KOA has received widespread attention. It was a common concern of global researchers to relieve the pain of KOA patients and restore the quality of life. Under the background that acupuncture accounts for a relatively high proportion, the next step may consider how to make the balanced development of a variety of Chinese non-pharmacological therapies. In addition, the problem of how to eliminate the placebo effect maybe the direction of future research.

## Introduction

Knee osteoarthritis (KOA) is a common chronic joint disease characterized by articular cartilage damage, osteophyte formation, and synovial hyperplasia accompanied by knee pain, and limited function. It was the osteoarthritis with the highest disability rate (Chen et al., 2021), with approximately 14 million patients suffering from KOA in the United States and facing high surgical costs (Deshpande et al., 2016). The Osteoarthritis Research Society International (OARSI) and the U.S. Department of Defense (DoD) have noted the benefits of non-surgical management for the treatment of KOA (Bannuru et al., 2019; Krishnamurthy et al., 2021), however, there is currently no treatment that can reverse the joint damage caused by KOA. KOA is mainly treated to relieve pain and improve joint mobility.

Traditional Chinese non-pharmacological therapies refers to a series of external therapies under the guidance of traditional Chinese medicine theory. Many studies support the benefits of traditional Chinese non-pharmacological therapies including acupuncture, Tai Chi, moxibustion, and many others, which not only improves pain, depression (Zhang and Yuan, 2020; Ho et al., 2021), and sleep quality (Lu et al., 2017), but also helps reduce the operation rates (Gang et al., 2020) and the financial burden on patients. A survey shows that acupuncture as an intervention method of KOA was expected to save 100,000 pounds per year (White A. et al., 2012). The 2014 guidelines for the management of hip and knee osteoarthritis by the National Institute of Health and Clinical Excellence (NICE) recommends non-pharmacological intervention as core intervention (National Institute for Health and Care Excellence, 2014). In summary, traditional Chinese non-pharmacological therapies is a safe, effective, and low side effect option.

Bibliometrics using quantitative methods to describe published studies according to a scientific cartography program that allows researchers to visually find out the evolution process and classical literature of a discipline. It provides an important quantitative basis for macroscopically understanding the key topics and research trends of a discipline. Recently an increasing number of bibliometric analyses have shown potential therapeutic effects of traditional Chinese non-pharmacological therapies in facial palsy (Zhang X. et al., 2012), post-stroke rehabilitation (Sun et al., 2012), cognitive impairment (Li et al., 2021), and cardiac disease (Li et al., 2022), suggesting that bibliometric has been widely used in clinical and basic studies. However, a bibliometric analysis of the use of traditional Chinese non-pharmacological therapies in KOA has not been conducted as far as we know. Therefore, the purpose of this work was to use bibliometric visualization tools to analyze the research status, hotspots, and future trends of Chinese non-pharmacological therapies used in KOA treatment from 2012 to 2022, the results will be displayed in the form of knowledge map.

## Materials and methods

From September 2012 to 2022, literature related to the use of traditional Chinese non-pharmacological therapies in KOA was searched in the core database of the Web of Science and PubMed database. The data were searched according to “Knee Osteoarthritis” and “traditional Chinese non-pharmacological therapy,” and the specific retrieval formula is shown in Table 1. Data retrieval was not limited to the literature type and language. Initially 546 papers were obtained. Then, the repeated literature were further deleted manually, and only the literature related to the treatment of KOA with Chinese non-pharmacological therapy were included. Data collection and analysis were done independently by YL, SJ and MZ, and any differences were resolved through discussion or seeking the help of other authors. Finally, 375 articles were included.

**TABLE 1:** Search queries.

Set	Result	Search query
#1	36,538	{TS = [(Knee Osteoarthritis) OR (gonarthritis) OR (gonitis)]}
#2	15,010	{TS = [(baduanjin) OR (qigong) OR (taichi) OR (acupuncture) OR (tuina) OR (Acupotomy) OR (electro-acupuncture) OR (auricular needle) OR (Moxibustion) OR (acupoint) OR (catgut embedding) OR (Yi Jin Jing) OR (acupressure) OR (cupping therapy) OR (fuming-washing therapy) OR (needle knife therapy) OR (wax therapy) OR (bloodletting therapy) OR (intermediate frequency therapy) OR (gua sha)]}
#3	546	Indexes = WoS Core Collection, PubMed
		Editions = SCI Expanded–1900-present
		Timespan = 2012–2022
		#1 AND #2

CiteSpace (version6.1.R3) is a software designed to identify the scientific literature and present new trends and developments in the discipline (Chen and Song, 2019). It presents the structure, rule and distribution of subject knowledge in the form of scientific knowledge map that allows the discovery of advances and research frontiers in a given field. VOSviewer (Version1.6.14) is a software for building visual bibliometric networks, constructed by a team of Leiden University (van Eck and Waltman, 2010). Using VOSviewer and SCImago Graphica, we completed studies on national geographic distribution, publication trends of literature, commonly used acupoints, evaluation indexes, and intervention methods. In order to ensure consistency, the top 50 of each element were selected for analysis. The results will be systematically reviewed in accordance with PRISMA guidelines.

## Results

### Number of published literature and annual trends

**FIGURE 1:**
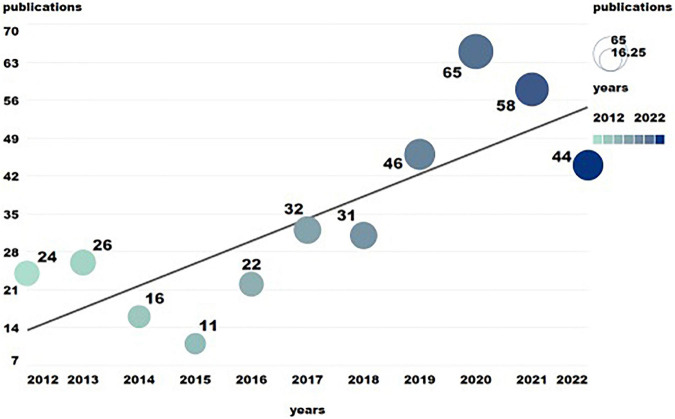
The annual number of publications related to traditional Chinese non-pharmacological therapies on knee osteoarthritis (KOA).

From 2012 to 2022, the number of literature on traditional Chinese non-pharmacological therapies in the treatment of KOA showed an overall fluctuating trend, with 375 literature published, or an average of 34 literature per year. After 2019, the number of publications increased significantly. In the same year, China promulgated the “opinions on promoting the inheritance and innovative Development of Traditional Chinese Medicine,” clearly pointing out that to promote the inheritance, openness and innovative development of TCM (Huang, 2019), the number of literature on KOA and other TCM treatments was expected to continue to grow in the future (Figure 1).

### Document type analysis

There were five types of literature in total, of which, 268 “Articles” were the largest type of literature (71.4%), indicating that a large number of clinical and basic trials have been conducted in the past decade to verify the efficacy of traditional Chinese non-pharmacological therapies in the treatment of KOA. This was followed by “Review” with 82 articles, indicating that retrospective research was also valued by researchers, while 25 articles (6.5%) were of other types (Table 2).

**TABLE 2:** Document types related to Chinese non-pharmacological therapy on KOA.

Ranking	Type	Counts (%)
1	Article	268 (71.4%)
2	Review	82 (21.8%)
3	Meeting abstract	13 (3.4%)
4	Editorial material	7 (1.8%)
5	Letter	5 (1.3%)

### National analysis

**FIGURE 2:**
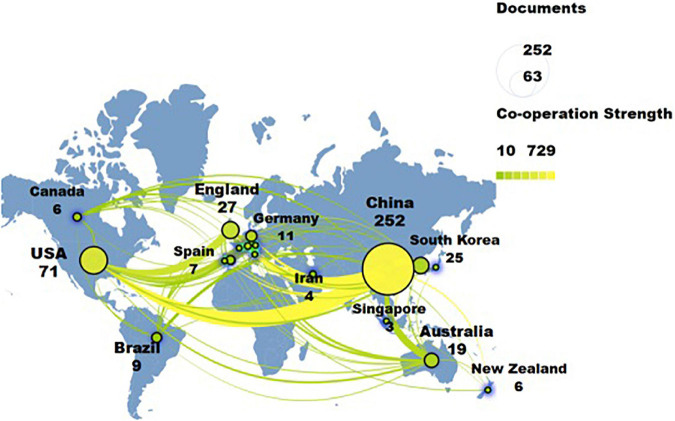
The collaboration network of countries researching traditional Chinese non-pharmacological therapies on knee osteoarthritis (KOA).

According to the national analysis, a total of 32 countries involved in the research of traditional Chinese non-pharmacological therapies on KOA from 2012 to 2022. China published 252 articles and was the most active country, the United States was the second (71), England (Wang et al., 2021) and South Korea (Vickers et al., 2012). Among them, China and the United States had the strongest correlation, indicating close cooperation among the regions. From the perspective of centrality, the United States ranked first (0.68), followed by Australia (0.21) and China (0.19). The top three most-cited countries were the United States (2363), China (1688), and England (1664; Table 3). Regionally, Asia and Europe were the research centers, with a clear clustering phenomenon. For example, China, Japan, South Korea, and Singapore were the major contributors to Asian research, while Spain, England, and Germany were the main drivers of European research (Figure 2).

**TABLE 3:** Top 10 countries related to Chinese non-pharmacological therapy on KOA.

Rank	Publications	Countries	Centrality	Countries	Citations	Countries
1	252	China	0.68	USA	2363	USA
2	71	USA	0.21	Australia	1688	China
3	27	England	0.19	China	1664	England
4	25	South Korea	0.14	England	1218	Germany
5	19	Australia	0.13	Spain	434	Swizerland
6	11	Germany	0.03	Brazil	407	Australia
7	9	Brazil	0.02	Germany	227	South Korea
8	7	Spain	0.02	Canada	194	Canada
9	6	Canada	0.01	Iran	129	Spain
10	4	Swizerland	0.01	Italy	99	Brazil

### Institutional analysis

**FIGURE 3:**
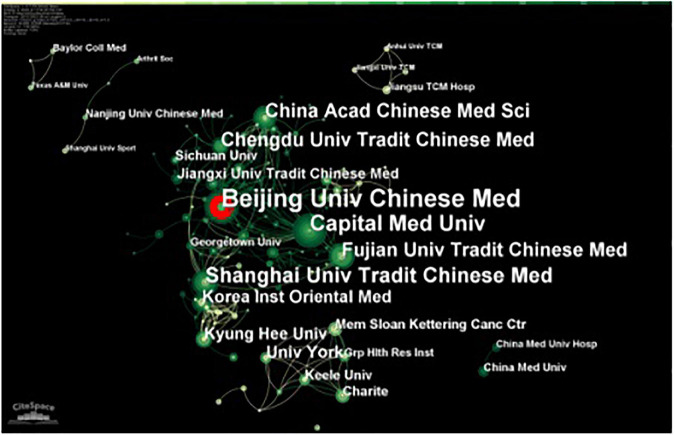
The collaboration map of institutions related to traditional Chinese non-pharmacological therapies on knee osteoarthritis (KOA).

According to the analysis of 557 institutions participating in studies of traditional Chinese non-pharmacological therapies on KOA over the past decade, 36 institutions published more than five articles (Figure 3), with Beijing University of Chinese Medicine (Tu et al., 2021) published the most, followed by Shanghai University of Chinese Medicine (National Institute for Health and Care Excellence, 2014), and Capital Medical University (White A. et al., 2012), which noted that traditional Chinese non-pharmacological therapies helped to improve pain and restore the knee function in KOA patients (Wang M. et al., 2020; Zhang Y. et al., 2020; Tu et al., 2021), suggesting that this effect may be achieved through the changes of inflammatory cytokines TNG-α, IL-1β, and IL-13 (Shi et al., 2020), which have received more attention in recent years. In terms of centrality, Beijing University of Chinese Medicine (0.1) ranked the top one, followed by Capital Medical University (0.1), and Korea Medical College of Oriental Medicine (0.08). The top three most frequently cited universities were University of York (1314), University of Southampton (1237), and Memorial Sloan-Kettering Cancer Center (1185, Table 4). Through the close collaboration with Klee University and Charite, their study indicated that the effect of acupuncture on KOA was related to the amount and duration of acupuncture (MacPherson et al., 2013; Vickers et al., 2018), and the effect was long-lasting (MacPherson et al., 2017), suggesting that acupuncture was a recommended therapy for KOA (Vickers et al., 2012).

**TABLE 4:** Top 10 institutions related to Chinese non-pharmacological therapy on KOA.

Rank	Publications	Institutions	Centrality	Institutions	Citations	Institutions
1	20	Beijing Univ Chinese Med	0.1	Beijing Univ Chinese Med	1314	Univ York
2	10	Shanghai Univ Tradit Chinese Med	0.1	China Acad Chinese Med Sci	1237	Univ Southampton
3	9	Capital Med Univ	0.08	Korea Inst Oriental Med	1185	Mem Sloan Kettering Canc Ctr
4	9	Chengdu Univ Tradit Chinese Med	0.07	Capital Med Univ	1102	Keele Univ
5	8	China Acad Chinese Med Sci	0.06	Fujian Univ Tradit Chinese Med	505	Charite
6	8	Fujian Univ Tradit Chinese Med	0.05	Shanghai Univ Tradit Chinese Med	435	Univ Maryland
7	8	Univ Hong Kong	0.04	Harvard Med Sch	269	Univ Melbourne
8	7	Korea Inst Oriental Med	0.04	Chengdu Univ Tradit Chinese Med	248	Beijing Univ Chinese Med
9	7	Kyung Hee Univ	0.03	Univ Hong Kong	223	Massachusetts Gen Hosp
10	6	Shanghai Jiao Tong Univ	0.02	Massachusetts Gen Hosp	193	China Acad Chinese Med Sci

### Author analysis

**FIGURE 4:**
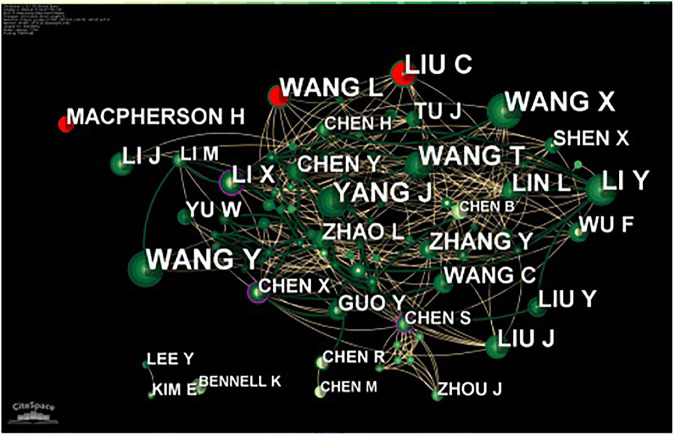
The collaboration map of Authors related to traditional Chinese non-pharmacological therapies on knee osteoarthritis (KOA).

Over the past decade, 2,021 authors participated in the study of traditional Chinese non-pharmacological therapies for KOA, among which, 27 authors have published more than five articles. The three authors with the most published literature were Cun-Zhi Liu (Li et al., 2021), Jian-Feng Tu (Li et al., 2021), and Li-Qiong Wang (Zhang X. et al., 2012), who concluded that acupuncture was an effective method by observing specific changes in the brain function in patients with KOA (Zhang N. et al., 2020). They also found that intestinal flora was a possible target (Wang et al., 2021), and improving the frequency of treatment may lead to better outcomes (Lin et al., 2020). The three authors with the highest centrality were Wang X (0.11), Li X (0.08), and Li Y (0.08). The most frequently cited author was Lewith G (1153; Table 5), who draws a negative conclusion on traditional Chinese non-pharmacological therapies, suggesting that acupuncture and Qi Gong were not particularly effective compared to controls and that patient’s confidence in the physician and treatment method had a significant effect on the outcome (Macfarlane et al., 2012; White P. et al., 2012). Macpherson H (1128) and Vickers A (1066) also had high citation frequency (Table 5). The authors with the purple outer circles indicate a high between centrality and were core researchers in the research field. The authors with the red mark represent a prominent frequency over a period of time. For example, Macpherson H, Cun-Zhi Liu, and Li-Qiong Wang were the most active authors in the last decade (Figure 4).

**TABLE 5:** Top 10 authors related to Chinese non-pharmacological therapy on KOA.

Rank	Publications	Author	Centrality	Author	Citation	Author
1	13	Cun-Zhi Liu	0.11	Wang X	1153	Lewith G.
2	13	Jian-Feng Tu	0.08	Li X	1128	Macpherson H.
3	11	Li-Qiong Wang	0.06	Li Y	1066	Vickers A.
4	10	Tian-Qi Wang	0.06	Lin L	988	Foster N.
5	10	Fan Wu	0.06	Chen S	985	Witt C.
6	10	Jing-Wen Yang	0.06	Chen X	979	Linde K.
7	9	Li-xing Lao	0.05	Liu J	979	Sherman K.
8	9	Lu-Lu Lin	0.05	Li J	718	Maschino A.
9	9	Xue-Yong Shen	0.05	Li B	641	Cronin A.
10	9	Ling Zhao	0.05	Wu F	275	Kong J.

### Journal analysis

**FIGURE 5:**
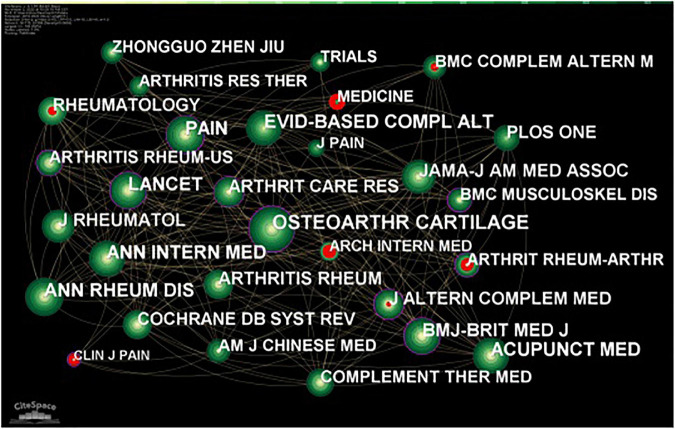
The co-cited journal related to traditional Chinese non-pharmacological therapies on knee osteoarthritis (KOA).

Journal analysis showed that a total of 2,994 journals were cited, of which 107 journals were cited more than 20 times. The most frequently cited journal in total was *Osteoarthritis Cartilage* (560), followed by *Pain* (409), and *Acupuncture in Medicine* (365). The top three journals in terms of centrality were *Arthritis Rheumatism* (0.1), *Arthritis Care Research* (0.08), and *Complementary Therapies in Medicine* (0.08). The top three journals in terms of number of publications were *Evidence-Based Complementary* and *Alternative Medicine* (Wang et al., 2016), *Medicine* (Choi et al., 2017), and *Trials* (Vickers et al., 2012; Table 6). Journals with purple outer rings indicate high between centrality, publish important literature and were key hubs for the entire network. Journals marked in red indicate a high highlighting frequency during a given time period. For example, *clinical Journal of Pain*, *Journal of Pain Research*, and *Medicine* were the journals that have been highlighted most frequently in the last 3 years (Figure 5).

**TABLE 6:** Top 10 co-cited journals related to Chinese non-pharmacological therapy on KOA.

Rank	Co-citation	Journal	Centrality	Journal	Publications	Journal
1	560	Osteoarthr Cartilage	0.1	Arthritis Rheum-US	40	Evid-Based Compl Alt
2	409	Pain	0.08	Arthrit Care Res	35	Medicine
3	365	Acupunct Med	0.08	Complement Ther Med	25	Trials
4	358	Evid-Based Compl Alt	0.08	J Altern Complem Med	20	Acupunct Med
5	318	ANN Rheum Dis	0.07	Clin J Pain	13	Osteoarthr Cartilage
6	313	ANN Intern Med	0.06	Lancet	13	J Tradit Chin Med
7	264	BMJ-Brit Med J	0.06	Zhen Ci Yan Jiu	11	J Pain Res
8	250	Lancet	0.06	Zhongguo Gu Shang	8	Plos One
9	214	J Alter Complem Med	0.05	Arthrit Care Res	7	BMJ Open
10	206	JAMA	0.05	Arthritis Res Ther	6	Int J Clin Exp Med

### Reference analysis

**FIGURE 6:**
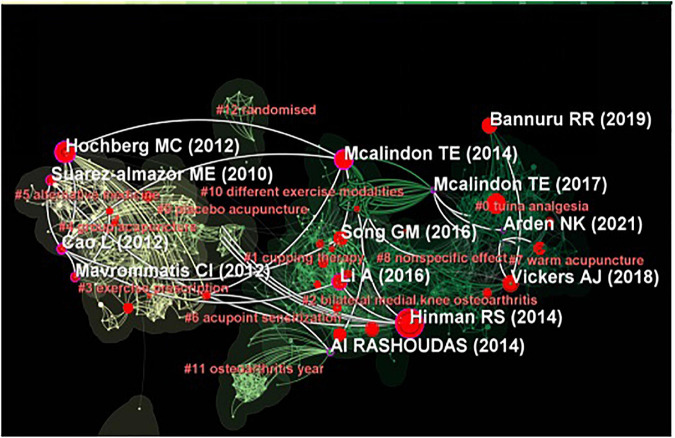
The network and cluster map of co-cited references related to traditional Chinese non-pharmacological therapies on knee osteoarthritis (KOA).

We screened the most representative literature about traditional Chinese non-pharmacological therapies in the treatment of KOA from 2012 to 2022. The yellow line represents literature from 2012 to 2015, and the representative cluster labels were “alternative therapy,” “group acupuncture” and “exercise prescription,” and the green line represents the literature from 2016 to 2022 with the representative cluster labels of “knee osteoarthritis,” “massage analgesia,” “warm acupuncture,” and “non-specific efficacy” (Figure 6). We found that the most frequently cited literature was the 2014 guidelines issued by the International Society for Osteoarthritis Research (OARSI), which listed Tai Chi as a core treatment for KOA (McAlindon et al., 2014). In addition, the American College of Rheumatology guidelines also list Tai Chi as a recommended treatment for KOA (Hochberg et al., 2012). These two guidelines have received extensive attention from researchers, and their recommendations have been adopted in a number of studies (Table 7). Six of the first 10 cited articles discussed the clinical effects of acupuncture on KOA, and the use of which has received a lot attention from researchers, particularly the placebo effect of acupuncture. A randomized controlled clinical trial published by Hinman RS in JAMA showed that acupuncture was no more beneficial than sham acupuncture in treating chronic knee pain (Hinman et al., 2014).

**TABLE 7:** Top 10 cited references related to Chinese non-pharmacological therapy on KOA.

Rank	Citations	Cited reference	Journal	Representative author (publication year)
1	1741	OARSI guidelines for the non-surgical management of knee, hip, and polyarticular osteoarthritis	Osteoarthritis Cartilage	McAlindon et al. (2014)
2	641	Acupuncture for chronic pain individual patient data meta-analysis	JAMA Intern Med	Vickers et al. (2012)
3	270	Acupuncture for chronic pain: Update of an individual patient data meta-analysis	J Pain	Vickers et al. (2018)
4	146	Acupuncture for chronic knee pain a randomized clinical trial	JAMA	Hinman et al. (2014)
5	106	Evidence-based evaluation of complementary health approaches for pain management in the United States	Mayo Clin Proc	Nahin et al. (2016)
6	102	Acupuncture and other physical treatments for the relief of pain due to osteoarthritis of the knee: Network meta-analysis	Osteoarthritis Cartilage	Corbett et al. (2013)
7	84	The persistence of the effects of acupuncture after a course of treatment: a meta-analysis of patients with chronic pain	PAIN	MacPherson et al. (2017)
8	77	Influence of control group on effect size in trials of acupuncture for chronic pain: A secondary analysis of an individual patient data meta-analysis	PLoS One	MacPherson et al. (2014)
9	76	Pain management with acupuncture in osteoarthritis: A systematic review and meta-analysis	BMC Coplem Altern M	Manyanga et al. (2014)
10	76	Practice, practitioner, or placebo? A multifactorial, mixed-methods randomized controlled trial of acupuncture	PAIN	White P. et al. (2012)

### Keyword analysis

**FIGURE 7:**
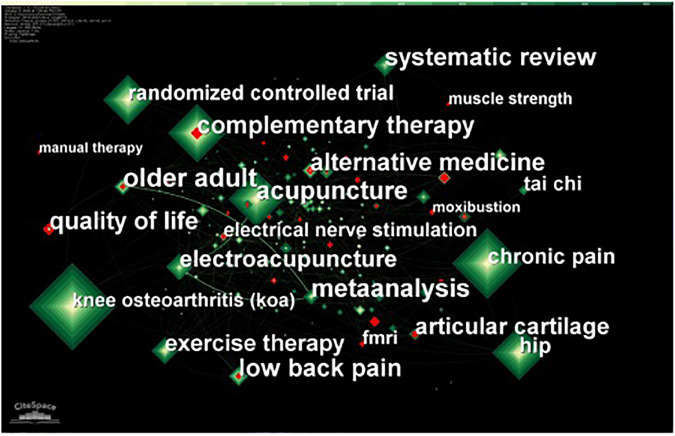
The network of co-occurrence keywords related to traditional Chinese non-pharmacological therapies on knee osteoarthritis (KOA).

We analyzed literature related to traditional Chinese non-pharmacological therapies on KOA in the past decade and summarized the keywords with the highest co-occurrence frequency and centrality (Table 8). The co-occurrence frequency indicates the frequency of the keywords, and the centrality represents the degree to which keywords are noticed. A node with a centrality greater than 0.1 indicates that the node has greater influence in the study. “Knee osteoarthritis” had the highest co-occurrence frequency (211), “adjunctive therapy” (172) and “pain” (160) were three keywords with the highest co-occurrence. The top three keywords in centrality were acupuncture (0.25), chronic pain (0.19), and alternative medicine (0.18). Figure 7 shows highlighted keywords with red nodes, representing great changes in co-occurrence frequency in a certain period of time and are used to find keywords that are decreasing or increasing. We found that “quality of life,” “older adult,” and “manual therapy” were the keywords that suddenly dominated the most during the period 2020–2022.

**TABLE 8:** Top 10 keywords related to Chinese non-pharmacological therapy on KOA.

Rank	Co-occurence	Keyword	Centrality	Keyword
1	211	Knee osteoarthritis	0.25	Acupuncture
2	172	Chronic pain	0.19	Chronic pain
3	160	Acupuncture	0.18	Alternative medicine
4	102	Hip	0.13	Adjunctive therapy
5	106	Osteoarthritis	0.12	Efficacy
6	89	Adjunctive therapy	0.1	Electro-acupuncture
7	85	Randomized controlled trial	0.08	Hip
8	78	Managment	0.08	Randomized controlled trial
9	58	Electro-acupuncture	0.08	Systematic review
10	43	OARIS recommendations	0.08	Older adult

**FIGURE 8:**
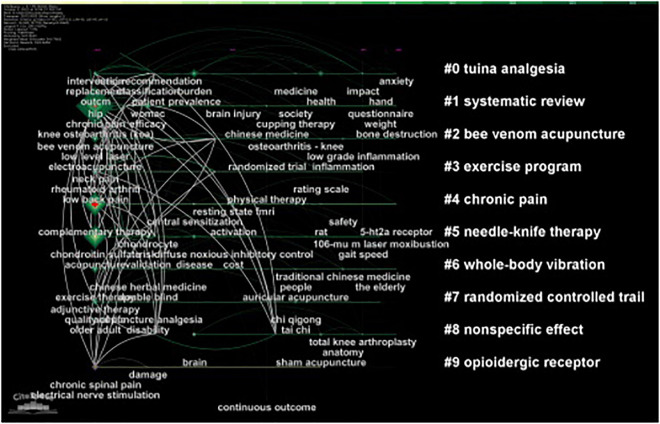
The timeline map of co-occurrence keywords related to traditional Chinese non-pharmacological therapies on knee osteoarthritis (KOA).

Figure 8 shows the keyword change and keyword clustering over time from 2012 to 2022. We found that the traditional Chinese non-pharmacological therapies such as “Tai Chi,” “Qi Gong,” “electro-acupuncture,” and “auricular acupuncture” will always be high-frequency keywords, and the emotion, inflammation and evaluation scales of KOA patients will also have higher co-occurrence frequency. Among the first 10 keyword clusters, “chronic pain” was the largest cluster with a total of 67 members.

### Analysis of therapies, acupoints, and evaluation index

We found that many traditional Chinese non-pharmacological therapies such as acupuncture and moxibustion involve the use of acupoints. In order to understand the use of acupoints in the treatment of KOA, we used VOSviewer and SCImago Graphica with co-occurrence frequency as screening condition. The most commonly used acupoints for treating KOA were analyzed. In addition, for other traditional Chinese non-pharmacological therapies, we analyzed the use frequency and corresponding evaluation indexes in the same way, hoping to provide ideas and references for researchers and clinicians.

**FIGURE 9:**
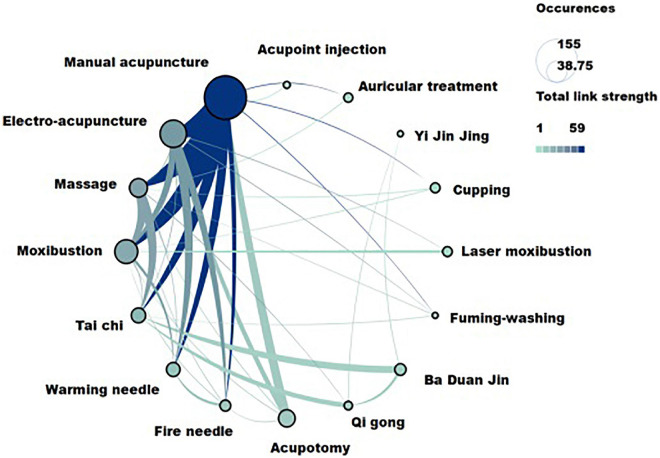
The therapies with co-occurrence frequency greater than five related to traditional Chinese non-pharmacological therapies on knee osteoarthritis (KOA).

**FIGURE 10:**
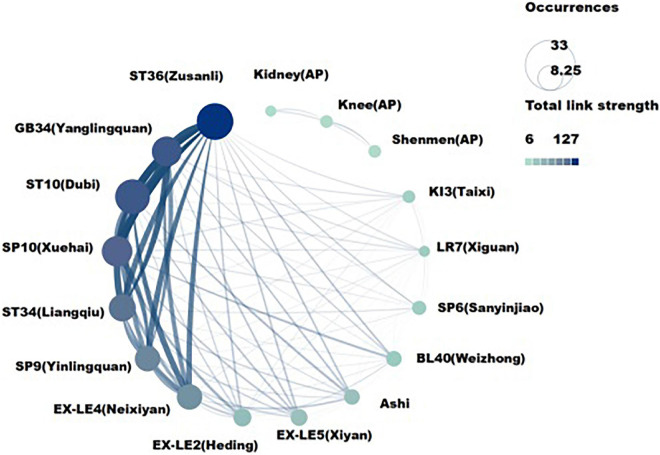
The acupoints with co-occurrence frequency greater than five related to traditional Chinese non-pharmacological therapies on knee osteoarthritis (KOA).

**FIGURE 11:**
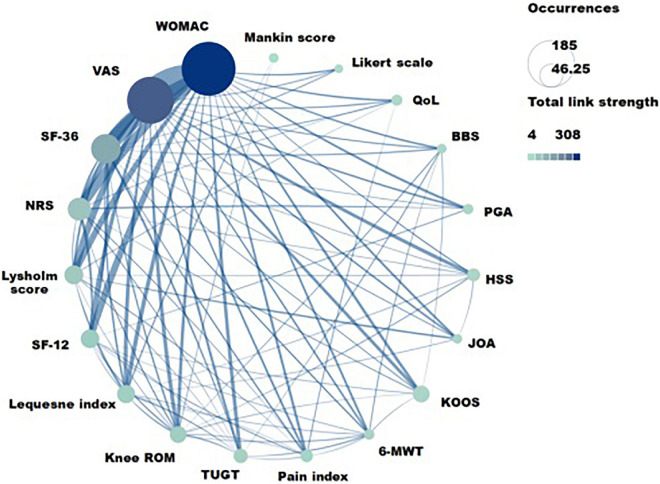
The evaluation indexes with co-occurrence frequency greater than five related to traditional Chinese non-pharmacological therapies on knee osteoarthritis (KOA). WOMAC, The western Ontario and McMaster universities osteoarthritis index; VAS, Pain visual analog scale; NRS, Numeric rating scale; Kee ROM, Knee range of motion test; TUGT, Timed up and go test; 6-MWT, 6-Minute walk test; KOOS, The knee injury and osteoarthritis score; JOA, Japanese Orthopedic Association knee score; HSS, Hospital for special surgery knee score; PGA, Patient Global Assessment; BBS, Berg balance scale; QoL, Qualiy of life.

The results showed that a total of 22 Chinese non-pharmacological interventions were used to treat KOA, and 16 interventions were co-occurring more than five times as often (Figure 9). A total of 49 acupoints have been mentioned in the article, 17 of which had a co-occurrence frequency of more than three times (Figure 10), and a total of 112 evaluation indexes were mentioned. Of these, 19 evaluation indicators had a co-occurrence frequency of more than five times (Figure 11).

The three most frequently used acupoints were ST36 (Zusanli), ST10 (Dubi), and GB34 (Yanglingquan). The three most widely used intervention methods were manual acupuncture, electro-acupuncture, and moxibustion, and the three most widely used evaluation indicators were WOMAC, VAS, and SF-36 (Table 9).

**TABLE 9:** Top 10 co-occurence indexes, therapies, and acupoints related to Chinese non-pharmacological therapy on KOA.

Rank	Co-occurence	Evaluation index	Co-occurence	Therapies	Co-occurence	Acupoints
1	185	WOMAC	155	Manual acupuncture	33	ST36 (Zusanli)
2	140	VAS	61	Electro-acupuncture	30	ST10 (Dubi)
3	55	SF-36	46	Moxibustion	23	GB34 (Yanglingquan)
4	34	NRS	28	Massage	22	SP10 (Xuehai)
5	23	Lysholm score	24	Acupotomy	18	ST34 (Liangqiu)
6	22	SF-12	18	Tai Chi	16	EX-LE4 (Neixiyan)
7	21	Lequesne index	16	Warming needle	8	EX-LE2 (Heding)
8	18	Knee ROM	11	Ba Duan Jin	7	EX-LE5 (Xiyan)
9	13	TUGT	10	Fire needle	6	SP9 (Yinlingquan)
10	10	Pain index	8	Cupping	6	Ashi

## Discussion

This study aims to describe the global participation and research trends of traditional Chinese therapies in the treatment of KOA. traditional Chinese non-pharmacological therapies, the benefits of which were gradually being recognized globally, may be a potential treatment for KOA. As the birthplace of traditional Chinese medicine culture, China has a large number of researchers participating in this field. It should be noted that despite the fact that Chinese scientists published the most academic findings, academic publications had little effect, which may be related to the rapid increase in the number of literature in recent years and the lack of domestic academic cooperation. South Korea and Japan were influenced by traditional Chinese medicine (TCM) since the Tang dynasty (Yu et al., 2017). Korea Institute of Oriental Medicine and Kyung Hee University as representative research institutions have made active exploration on the treatment of KOA (Choi et al., 2017, 2021). Western developed countries were also inclined to TCM which has been incorporated into regulations in some countries (Xue et al., 2009; Luo et al., 2018). The International Society for Osteoarthritis Research (OARSI) recommends that acupuncture should be added to the treatment of knee osteoarthritis (Bannuru et al., 2019). National legislation and the introduction of international guidelines has promoted the research in western countries to some extent.

Acupuncture was the most widely used Chinese non-pharmacologic therapy, among which ST36 (Zusanli) was the most frequently used acupoint, while most other acupoints were concentrated near the knee joint, reflecting the distribution of pain sensitivity (Luo et al., 2018). A lot of studies have demonstrated that acupuncture can help relieve pain in patients with KOA, promote recovery and reduce the burden on patients and society (Wang et al., 2016; Ma et al., 2017). Traditional Chinese non-pharmacological therapies such as moxibustion and Tai Chi have also shown high credibility (Liu et al., 2017; Xiao et al., 2020; Guo et al., 2022). The International Society for Osteoarthritis (OARSI) even recommends Tai Chi for all knee osteoarthritis patients because of its satisfactory performance in relieving pain and improving proprioception of knee joints (Woods et al., 2017; Liu et al., 2019; Hu et al., 2020). For the efficacy of traditional Chinese non-pharmacological therapies, researchers generally used WOMAC to evaluate knee joint dysfunction because of its objectivity, effectiveness, and sensitivity (Wolfe, 1999; Lingard et al., 2001; Da et al., 2021). The quality of life and emotion of elderly patients have been paid much more attention in recent years, and the majority of them were female, accounting for about 65% of KOA patients, with an average age of about 65 years (Li et al., 2018; Wang Q. et al., 2020; Zhao et al., 2021), which may be related to post-menopausal osteoporosis (Zhang Y. et al., 2012; Qin et al., 2013). Notably, certain randomized controlled clinical trials have revealed subpar evidence of traditional Chinese non-pharmacological therapy’s efficacy against KOA (Li et al., 2017; Lam et al., 2021), and the National Institute for Health and Care Excellence (NICE) recommendations even advise against the use of acupuncture for osteoarthritis (Conaghan et al., 2008). Therefore, more randomized controlled trials with strict design are needed to confirm the impact of traditional Chinese non-pharmacological therapies on KOA.

## Limitations

Citespace software can not directly identify literature from PubMed database, we need to convert the PubMed data into WoS format, which may lead to identification bias of some data, we have tried our best to correct this situation, but there may still be minor deviations.

## Conclusion

Chinese non-pharmacological therapies has shown certain advantages in the treatment of KOA and has received wide attention. Relieving pain and restoring quality of life of KOA patients has been a common concern of researchers all over the world. At present, the development of Chinese non-pharmacological therapies was unbalanced with acupuncture accounting for a relatively high proportion, and the application of tuina, moxibustion, and other therapies in KOA needs to be further explored. In addition, how to exclude the possible placebo effect in response to international doubts is also an urgent problem for the future.

## Author contributions

XT and XZ contributed to the concept and design of the research. MZ, YL, and SJ conducted the data collection and analysis. SZ and YW wrote the first draft of the manuscript. All authors contributed to the revision of the manuscript, read, and approved the submitted version.
